# Location-specific signatures of Crohn’s disease at a multi-omics scale

**DOI:** 10.1186/s40168-022-01331-x

**Published:** 2022-08-24

**Authors:** Carlos G. Gonzalez, Robert H. Mills, Qiyun Zhu, Consuelo Sauceda, Rob Knight, Parambir S. Dulai, David J. Gonzalez

**Affiliations:** 1 Department of Pharmacology, University of California San Diego, San Diego, CA 92093 USA; 2 Department of Pediatrics, University of California San Diego, San Diego, CA 92093 USA; 3 Department of Medicine, Division of Gastroenterology and Hepatology, Feinberg School of Medicine Northwestern University, Chicago, IL 60061 USA; 4 School of Pharmacy and Pharmaceutical Sciences, University of California San Diego, San Diego, CA 92093 USA; 5 Center for Microbiome Innovation, University of California San Diego, San Diego, CA 92093 USA; 6 Department of Bioengineering, University of California San Diego, San Diego, CA 92093 USA; 7 Department of Computer Science & Engineering, University of California San Diego, San Diego, CA 92093 USA; 8 School of Life Sciences, Arizona State University, Tempe, AZ USA; 9 Biodesign Center for Fundamental and Applied Microbiomics, Arizona State University, Tempe, AZ USA

**Keywords:** Inflammatory bowel disease, Crohn’s disease, Ileal Crohn’s, Colonic Crohn’s, Microbiome, Multi-omics

## Abstract

**Background:**

Crohn’s disease (CD), an inflammatory bowel disease (IBD) subtype, results from pathologic interactions between host cells and its resident gut microbes. CD manifests in both isolated disease locations (ileum or colon) or a combination of locations (ileocolonic). To date, a comprehensive understanding of how isolated CD subtypes influence molecular profiles remains outstanding. To address this, we sought to define CD location signatures by leveraging a large cross-sectional feature set captured from the stool of over 200 IBD patients and healthy controls using metaproteomics, shotgun metagenomics, 16S rRNA sequencing, metabolomic profiling, and host genetics paired with clinical endoscopic assessments.

**Results:**

Neither metagenomic nor host genetics alone distinguished CD location subtypes. In contrast, ileal and colonic CD were distinguished using mass spectrometry-based methods (metabolomics or metaproteomics) or a combined multi-omic feature set. This multi-omic feature set revealed colonic CD was strongly associated with neutrophil-related proteins. Additionally, colonic CD displayed a disease-severity-related association with *Bacteroides vulgatus*. Colonic CD and ulcerative colitis profiles harbored strikingly similar feature enrichments compared to ileal CD, including neutrophil-related protein enrichments. Compared to colonic CD, ileal CD profiles displayed increased primary and secondary bile acid levels and concomitant shifts in taxa with noted sensitivities such as *Faecalibacterium prausnitzii* or affinities for bile acid-rich environments, including *Gammaproteobacteria* and *Blautia* sp. Having shown robust molecular and microbial distinctions tied to CD locations, we leveraged these profiles to generate location-specific disease severity biomarkers that surpass the performance of Calprotectin.

**Conclusions:**

When compared using multi-omics features, colonic- and ileal-isolated CD subtypes display striking differences that suggest separate location-specific pathologies. Colonic CD’s strong similarity to ulcerative colitis, including neutrophil and *Bacteroides vulgatus* involvement, is also evidence of a shared pathology for colonic-isolated IBD subtypes, while ileal CD maintains a unique, bile acid-driven profile. More broadly, this study demonstrates the power of multi-omics approaches for IBD biomarker discovery and elucidating the underlying biology.

Video Abstract

**Supplementary Information:**

The online version contains supplementary material available at 10.1186/s40168-022-01331-x.

## Introduction

Inflammatory bowel disease (IBD) consists of two major subtypes, Crohn’s disease (CD) and ulcerative colitis (UC), with CD further sub-categorized into several subtypes including ileal (ICD), ileocolonic (ICCD), and colonic CD (CCD). Although these conditions are all classified as IBD, they harbor important differences in epidemiology, genetic abnormalities, clinical presentation, treatment effectiveness, and long-term complications [1–4]. Research in the past decades has shown host-gut microbe interactions influence many of these factors in clinically relevant ways [5–7]. Indeed, intestinal microbiota are increasingly recognized for their potential as IBD biomarkers and treatment targets, yet in-depth knowledge of how differences in microbe-host interactions shape CD location subtypes is lacking [8, 9]. This is largely due to limitations such as small patient cohorts, limited metadata, reliance on patient-reported indices in place of endoscopic measurements, and a lack of comprehensive host-gut microbe profiling [10–13].

To date, the largest IBD multi-omic profiling effort enrolled approximately 117 total subjects, with 67 CD patients included [13]. However, profiling CD’s inherent heterogeneity would benefit from a large number of cross-sectional samples and multiple profiling methods to accurately characterize categories such as disease location. Therefore, a need remains for a large-scale study leveraging multi-omic approaches that focuses on revealing the molecular underpinnings of CD location subtypes through the lens of host and gut-microbe interactions. We hypothesized this approach could help reveal the biological rationale driving differences in CD location and further provide the basis for disease-severity biomarkers tailored to specific CD locations (e.g., ICD and CCD) [14].

To test this, our group recently generated an expansive multi-omics feature set consisting of fecal 16S rRNA gene amplicon sequencing (ASVs), shotgun metagenomics, metabolomics, and metaproteomics from healthy controls and IBD patients spanning all major CD subtypes and severities [15]. These efforts were further supported by single-nucleotide polymorphism (SNP) sequencing of IBD patients covering known IBD-related mutations. By leveraging these feature sets, we reveal that colonic-isolated IBD subtypes (CCD and UC) are enriched in neutrophil-related proteins and a unique disease severity related association with the taxon *Bacteroides vulgatus*. In contrast, ICD is largely distinguished by increased bile acid levels along with alternations in taxa with known associations with bile acids (both sensitivity to and affinity for). Given the evidence for a robust location-specific fingerprint, we provide guidance on location-specific disease severity biomarkers that outperforms the current gold standard clinical biomarker, Calprotectin. Together, our results highlight the power of profiling complex phenotypes with multiple-omics types.

## Results

### Disease location, severity, and microbial diversity influence overall subject profiles

**Fig. 1:**
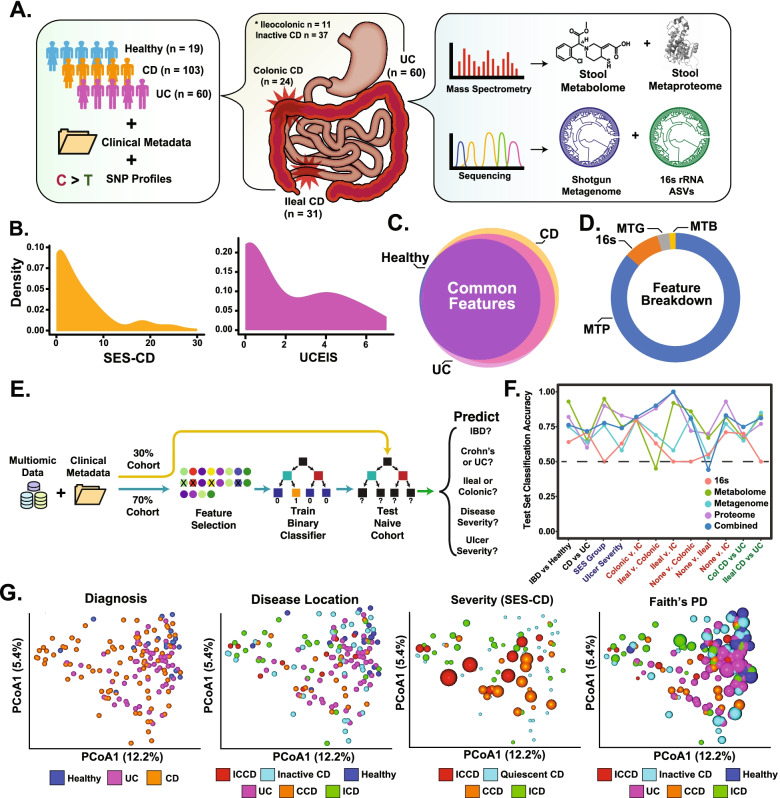
Cohort and high-level feature overview. **A** Cohort and pipeline overview. **B** Density plot of disease severity, as measured by either SES-CD for CD (left) or Ulcerative Colitis Endoscopy Index of Severity (UCEIS, right) for UC. **C** Venn diagram of all features overlap from our cohort. Common features = 6.022, unique CD = 927, unique UC = 454. **D** Relative proportion each feature set contributed to the multi-omic feature set. Of note, only OGUs were used in this analysis, and thus percentage would change based on sequence annotation workflow. **E** Schematic of machine-learning pipeline. **F** Test set classification accuracy scores for individual feature sets and the multi-omic (combined) feature set across several different metadata categories. Expanded metrics (e.g., precision, recall, etc.) available in Supplementary Table S2. **G** Multi-omic-generated Bray-Curtis distance PCoA, colored by metadata category (listed above graph). Color scaling for SES-CD and Faith’s phylogenetic diversity (Faith’s PD) is continuous from minimal value (small dot) to maximum value (large dot)

Two hundred ten patient samples were initially subjected to multiple-omics pipelines and contributed to the features identified. After accounting for metadata completeness, we identified 182 subjects (103 CD, 60 UC, 19 healthy controls) as our core analysis cohort (Fig. 1A). A subset of 126 IBD patients were further assessed using single-nucleotide polymorphism (SNP) arrays. Each IBD subject had detailed metadata and paired endoscopic assessments taken at the time of stool and DNA collections. Patients were largely balanced between ICD and CCD, with representation of disease severity spectrum based on endoscopic indices (Fig. 1B, Supplementary Figure S1A). Importantly, most features identified in this cohort were quantified in every disease subset, increasing statistical comparison potential (Fig. 1C).

Over 125,000 features from five different -omic platforms were initially identified (Fig. 1D). 16S amplicon sequence variant (ASVs) identified with 1799 taxon (in > 1 sample). Bolstering these results, shotgun metagenomic sequencing further identified 3568 Operational Genomic units (OGU) using the Woltka taxonomy pipeline [16]. Leveraging feature-based molecular networking and the available public libraries for metabolite classification (GNPS), we identified 1,929 metabolomic features from untargeted mass spectrometry data [17]. Lastly, the feature set included 108,081 metaproteome features, which to date represents the deepest single-study metaproteome in the public domain (Supplementary Figure S1B). Of these features, 106,409 were microbial proteins, while 2031 were host-derived, similar to previous large-scale stool metaproteome ratios [18, 19].

Given our interest in regional CD differences, we first determined the ability of individual feature sets to distinguish CCD from ICD. Selecting 1206 SNPs with previously identified IBD associations, we observed no difference between ICD and CCD profiles, with near total overlap in PCA-generated confidence intervals, despite a difference in minor allelic frequency between ICD and CCD (CI = 0.95, Supplementary Figure S1C) [20]. ICD and CCD SNP diversity was similar, likely contributing to lack of differentiation. While this result does not discount the utility of individual SNPs (or subgroups of related SNPs) to influence host pathways and alter microbial communities (neither of which was analyzed in the paper to limit its scope), it suggests ICD and CCD stool-based profiles are more easily influenced by other features, limiting further SNP analyses for the purposes of this study. All single-omics feature sets significantly distinguished controls from UC and CD, similar to previous results, with mixed results on other categories (Bray-Curtis-based ß-diversity, PERMANOVA corrected, permutations = 999, *q* < 0.05, expanded PERMANOVA results for all data sets in Supplementary Table S1, Supplementary Figure S1D) [21, 22]. Intra-IBD comparisons using OGUs failed to differentiate UC from CCD (*q* > 0.05), while ASVs did (*q* = 0.005). However, neither ASV nor metagenomic feature sets differentiated isolated CD subtypes. In contrast, both metaproteomic and metabolomic feature sets, which inherently contain both microbe- and host-related features, significantly differentiated ICD and CCD profiles (*q* < 0.05), suggesting the inclusion of host features may be needed for subtle distinctions.

We next merged all feature sets (excluding SNPs) into a large multi-omic feature set to determine its ability to differentiate CD locations. To limit noise and spurious signals, we selected metagenomic, ASVs, and metabolomics features present in more than a single sample, while only including metaproteome features (298 host, 2344 microbial) quantified in all samples. This scheme was chosen to avoid suboptimal metaproteomic data imputation methods and focus analyses on highly abundant proteins. This filtering resulted in a total of 9937 features, a ~ 10× loss compared to initial features, but with the added benefit of substantially increasing its quantitative power. As a proof of feature set reliability, we first tested whether IBD-relevant metadata categories other than location (e.g., steroid or biologic use) distinguished healthy controls from IBD patients (an expected result). We observed all healthy vs. IBD comparisons were significant (*q* < 0.05), while intra-IBD comparisons using these categories were not (except for subjects that underwent surgical resection, Table 1). Similar to single feature sets, multi-omics features confirmed IBD subtypes were distinct from healthy controls and further distinguished CD from UC (*q* = 0.001) and isolated CD subtypes (ICD and CCD, *q* = 0.02), while ICCD was not significantly different to ICD and CCD, in line with its mixed-location phenotype. To further support these results, we generated a machine learning pipeline and tested each feature set’s ability to predict a variety of clinically relevant categories such as IBD subtype, disease location, severity group, and ulcer size (Fig. 1E). The results revealed that in the current study, mass spectrometry-generated features outperformed DNA-based methods in most categories, with the multi-omic feature set performing at a similar level to metaproteomics when focused on classifying ICD vs. CCD (Fig. 1F, expanded results in Supplementary Table S2).

**Table 1:** Relevant metadata group comparisons for significance

Category	Groups	Significance	Significant groups (*q* value)
Steroid	3	Y	Ctrl vs. unknown (0.003), Ctrl vs. yes (0.006)
Bowel_resec	2	Y	0.002
ICV_resec	2	Y	0.002
Smoker	3	N	NA
ASA	3	Y	Ctrl vs. unknown (0.002), Ctrl vs. yes (0.002)
AZA	3	Y	Ctrl vs. unknown (0.001), Ctrl vs. yes (0.001)
Sex	2	N	NA
Biologics	3	Y	Ctrl vs. unknown (0.003), Ctrl vs. yes (0.003)
Ulcer Group	2	N	NA

To investigate features driving global profile trends, we generated principal coordinate analysis (PCoA) plots (and associated results) overlayed with metadata features likely influencing ordination (e.g., significant differences in beta-diversity, Fig. 1G). The top 20-contributing PC1 features were largely dominated *γ-Proteobacteria* features (ASVs and OGUs, 55% of total features), while 40% of PC2 features were from the class *Clostridiales* (Supplementary Figure S1E, Supplementary Table S3). This suggests microbial features influence global profiles, but additional features are required to discriminate between more nuanced disease phenotypes such as ileal and colonic CD.

Together, these analyses suggest comprehensive multi-omic profiles illuminate subtle disease-related distinctions that single-omic feature sets miss. As such, we next explored how the feature sets interacted and uniquely contributed to disease location-based profiles.

### Colonic-related CD subtypes are dominated by increased host response and linked microbial and metabolic signatures

**Fig. 2:**
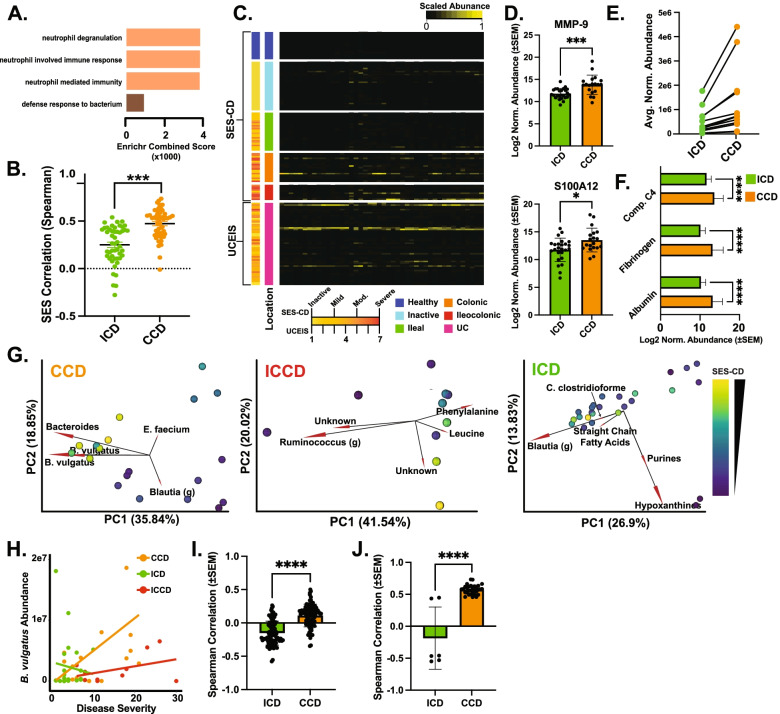
Multi-omics identifies features both common and unique features of ileal- and colonic-isolated diseases. **A** Enrichr-generated gene ontology enrichment graph using host proteome features significantly greater in CCD compared to ICD, sorted by Enrichr combination score [23]. **B** Comparison of neutrophil-associated proteins and SES-CD Spearman correlation scores, binned by CD location. Significance derived using Welch’s *t* test, *** = *p* < 0.0001. *n* = 49 features, all active disease ICD (*n* = 31) and CCD (*n* = 24) subjects used in comparison. **C** Heatmap of neutrophil-associated protein abundance (*x*-axis) compared in healthy controls, CD subtypes, and UC. Abundance is column scaled to enhance visibility. **D** Bar plot comparing log2-transformed relative protein abundance between ICD (*n* = 31) and CCD (*n* = 24). *p < 0.05, ***p < 0.0001, Welch’s *t* test. **E** Comparison of dipeptide/amino acid abundances between ICD (*n* = 31) and CCD (*n* = 24). Mean abundance levels plotted. Each dot represents a different amino acid/dipeptide identified and lines connecting dots denote the same metabolite in the disease location specified. **F** Comparison of blood-related proteins (complement C4, fibrinogen, and serum albumin) found in ICD (*n* = 31) and CCD (*n* = 24) stool samples. **** = *p* < 0.0001, Welch’s *t* test. **G** PCA biplot of individual CD locations, overlayed with SES-CD severity scores. Color ramping from purple to yellow denotes increasing severity score. Only five most contributing features (combined PCA1 + 2 contribution) plotted for ease of viewing. **H** Scatter plot comparing SES-CD scores (*x*-axis) and *B. vulgatus* metagenomic abundance. Linear model used to plot trend line. ICD *n* = 31, Rho = 0.29, *p* = 0.16; CCD *n* = 24, Rho = 0.48, *p* = 0.03; ICCD *n* = 11, Rho = 0.84, *p* = 0.07. **I** Bar plot comparing Spearman correlation values (*n* = 89 corr. pairs) of *B. vulgatus* levels and neutrophil related proteins, split by ICD and CCD. ****p < 0.0001, Welch’s *t* test. **J** Bar plot comparing significant (*p* < 0.05) Spearman correlation values of *B. vulgatus* levels and dipeptide metabolomic features, split by ICD (*n* = 6 features), and CCD (*n* = 31 features). ****p < 0.0001, Welch’s *t* test

Our high-level analyses revealed multi-omic profiles readily differentiated ICD and CCD, as such we next examined the features differentiating them. We observed the strongest enrichment in CCD subjects was generated by neutrophil degranulation-associated proteins, a trend confirmed even when controlling for severity score (Fig. 2A, Supplementary Figure S2A). In line with this observation, CCD patient SES-CD scores were significantly more correlated with their neutrophil-related protein abundance compared to ICD patients, even when only a single scored CCD segment (rectum) was used (Fig. 2B, Supplementary Figure S2B). To further control for both tissue involvement and disease severity, we further selected ICD (*n* = 13) and the rectal-only CCD patients (*n* = 6) with equal CD-SES scores (mean ICD = 6.1, CCD = 6.5) and compared their abundances (Supplementary Figure S2C). While this diminished the robustness of prior results, comparing the means of the top 10 proteins with the greatest difference revealed significantly greater levels in CCD, supporting the prior findings. UC patients also harbored significantly greater levels of neutrophil-related proteins compared to ICD despite having a similar distribution of patient severity scores when considering all active UC patients (UCEIS > 1 mean scaled score = 0.42, ICD SES-CD > 1 scaled score = 0.38, Fig. 2C) [24]. To reveal how inflammatory cytokines may influence the increased neutrophil activity in CCD, we imputed known inflammatory cytokines into protein-protein interaction networks generated by upregulated proteins in CCD (Supplementary Figure S2D, see Supplementary Table S4 for input list). This revealed a highly integrated network of inflammatory cytokines and proteins observed to be upregulated in CCD, with major imputed-observed connection hubs stemming from FN1, ITGAM, and ALB.

One consequence of neutrophil involvement is the secretion of proteases and inhibitors that alter proteolytic activity and drive disease severity. We observed protease-inhibitor pairs such as MMP-9 (Spearman ρ = 0.71, *p* = 0.0006) and S100A12 (Spearman ρ = 0.73, *p* = 0.0003), which can inactivate MMP-9, as correlated with disease severity in both CCD and ICD. However, the strength and significance of these correlations was decreased in ICD (Spearman ρ = 0.41, *p* = 0.04; Spearman ρ = 0.53, *p* = 0.005 respectively). Both proteins were significantly increased in CCD compared to ICD (Fig. 2D). In line with this, we observed generally increased levels of dipeptide and amino acid-related features in CCD (Fig. 2E, Supplementary Figure S2E). Since gut-based proteolytic activity is often linked to ‘gut leakiness’, we next searched our feature set for markers of possible blood infiltrate and observed the levels of proteins commonly found in blood such as serum albumin, compliment factors (C4), and fibrinogen were significantly higher in CCD subjects (Fig. 2F).

While the previous results reflect increased host-protease activity, these may solely reflect tissue injury and acute response and as such may not reflect a pathological increase in proteolytic activity. In contrast, many microbial proteases are known virulence factors [25, 26]. As such, we were also interested in microbial proteases contributed to this environment. We previously identified the taxa *Bacteroides vulgatus* (BV) contributed to UC pathogenesis, and this contribution was largely driven by protease overproduction [15]. Biplots of CD locations revealed BV also contributed to CCD severity ordination, but not ICD or ICCD (Fig. 2G). In agreement, relative BV levels in CCD subjects were significantly correlated with SES, despite non-significant differences in overall BV abundance between CD locations (Fig. 2H, Supplementary Figure S2F). BV was also positively correlated with neutrophil-related proteins in CCD yet displayed the opposite trend in ICD (Fig. 2I). Abundance of BV proteases was largely similar between CCD and ICD; however, BV proteases from CCD patients displayed a trend in overall positive association with disease severity (*p* = 0.06, Supplementary Figure S2G). Lastly, BV was strongly correlated with dipeptides in CCD patients but not in ICD, suggesting a potential BV-neutrophil proteolytic synergy that results in increased colonic proteolysis (Fig. 2J).

Overall, multi-omic profiling of CCD subjects revealed possible increases in neutrophil and microbially related proteolytic activity compared to ileal profiles.

### Alterations in metabolite levels heavily contribute to overall ICD profile

**Fig. 3:**
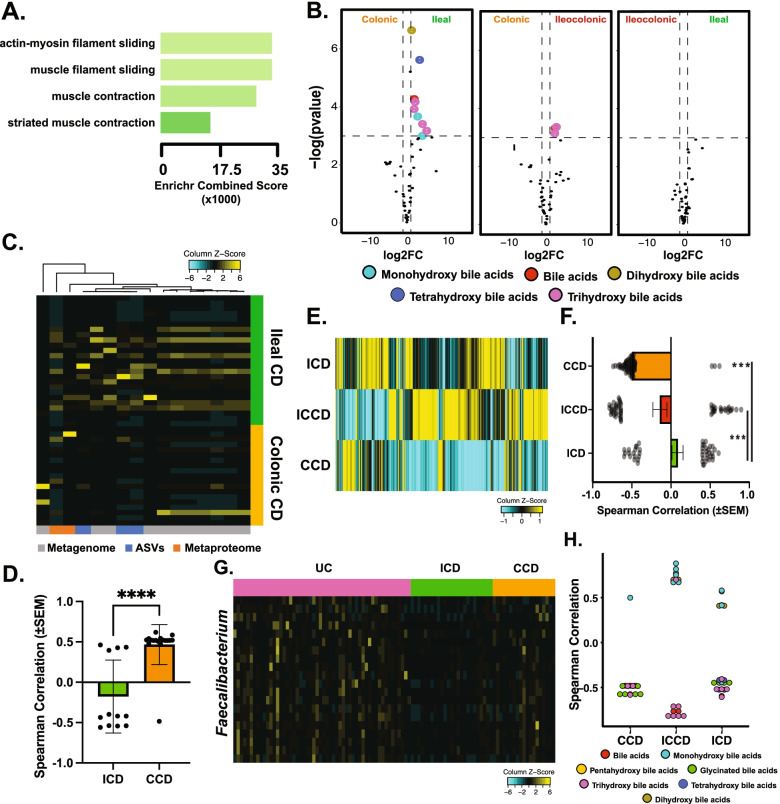
Multi-omics identifies features both common and unique features of ileal- and colonic-isolated diseases. **A** Enrichr-generated gene ontology enrichment graph using host proteome features significantly greater in ICD compared to CCD, sorted by Enrichr combination score [23]. **B** Volcano plots comparing major CD subtypes and the abundance of bile acid labeled metabolites. Significance cutoff = FDR < 0.05 and |log2 fold-change| > 0.5. Bile acid types denoted by specific colors. ICD *n* = 31, CCD *n* = 31, ICCD *n* = 11. **C** Heatmap of *Gammaproteobacteria* feature abundances (*x*-axis) in ICD (*n* = 31) and CCD (*n* = 24) subjects. Color key at bottom denotes feature-set origin. **D** Bar plot comparing Spearman correlation values (*Proteobacteria*-SES-CD, only significant values selected, see) between ICD (14 features) and CCD (17 features). **** signifies *p* < 0.0001, Welch’s *t* test. **E** Heatmap of *Lachnospiraceae* mean abundances for each CD subtype. Values scaled column-wise for visualization. **F** Bar plot comparing Spearman correlation values (*Lachnospiraceae*-SES-CD, only significant features [*p* < 0.05] selected, CCD = 88, ICD = 45, ICCD 56) between major CD subtypes. *** signifies *p* < 0.001, Welch’s *t* test. **G** Heatmap of *Faecalibacterium* feature abundance (*y*-axis, all feature sets included) among CD subtypes (*x*-axis). Feature abundances are row-scaled for emphasis. **H** Plot of *Faecalibacterium*-bile acids correlations, colored by subtype and split by location. Only significant features used in comparison

In contrast to CCD, clear immunogenic enrichments were largely absent in ICD, pointing instead towards increased levels of muscle-related proteins (Fig. 3A). These proteins were almost exclusively various forms of myosin. A similar trend was also observed when ICD was compared to UC patients, suggesting it is a common distinction between ileal- and colonic-involved subtypes. The enriched myosin proteins were not significantly associated with disease severity, ulcer severity, or the presence of strictures or penetrating wounds. However, comparing patients with any strictures or penetrating wounds (*n* = 10) to those without (*n* = 9) revealed a significant (*p* < 0.05) difference between these two groups, with increased abundance in patients with strictures or wounds (Supplementary Figure S3A). This difference was not as pronounced as overall comparisons to CCD, suggesting they may also be a generalized feature of ICD. Similar to our prior analysis, we next generated a cytokine inference protein-protein network map from proteins upregulated in ICD to reveal any possible connections to common inflammatory cytokines (Supplementary Figure S3B, Supplementary Table S4). In contrast to CCD, these cytokines were entirely segregated from the input network, with a single connection from TPM4 to IL2.

Next, we observed both primary and secondary bile acids displayed a robust increase in ICD patients compared to CCD, in line with known defects in bile acid reabsorption in ICD (Fig. 3B, Supplementary Table S5). Confirming ileal involvement is a major determinant of stool bile acids levels in CD, ICCD also harbored significantly altered levels of bile acids compared to CCD. Interestingly, bile acids were not broadly correlated with disease severity for any CD subtype, possibly suggesting their impact on severity is indirect, such as influence of microbial compositions.

Comporting with these findings, we observed a striking enrichment in *γ-Proteobacteria* (and *Proteobacteria* in general) levels in ICD, echoing prior reports [27] (Fig. 3C). While significant *Proteobacteria*-disease severity (SES-CD) correlations were present in both ICD and CCD, the strength and abundance of positive correlations was significantly greater in CCD (Fig. 3D), suggesting *Proteobacteria* are entrenched in ICD microbial communities, while their presence in CCD signifies active inflammation. After initially filtering for metabolites present in > 40% of samples (to increase correlational pairs), we observed ICD patient *Proteobacteria* levels were associated with bile acids and their derivatives, echoing prior evidence suggesting *Proteobacteria* are often resistant to antimicrobial effects of bile acids [28]. 40% of positively correlated *Proteobacteria*-metabolite pairs consisted of dihydroxylated bile acid, the strongest likely being isoursodeoxycholic acid (Supplementary Table S6). Relaxing this filter expanded the associations to primary bile acids as well (tri and tetrahydroxylated); however, their specific identity was most often unknown due to lack of annotation.

The family *Lachnospiraceae* (specifically *Ruminococcus* and *Blautia*) also distinguished ileal-involved CD subtypes from CCD (Fig. 3E, Supplementary Figure S3D). When correlated to disease severity, ileal-involved subtypes (ICD and ICCD) exhibited approximately equal positively and negatively correlated features, while CCD was comprised of nearly all negatively correlated features (Fig. 3F). *Blautia* sp. have previously been reported as processors of primary bile acids such as cholic acid [29]. In line with this, mono- and di-hydroxyl bile acids (e.g., secondary bile acids) were positively correlated with *Blautia* levels, while a tri- and tetra-hydroxyl (primary) bile acids exhibited the opposite trend (Supplementary Figure S3E).

Further supporting the role of bile acids in shaping ICD’s microbial-community structure, our machine learning pipeline identified *Faecalibacterium prausnitzii (F. prausnitzii)* as highly discriminative of ileal- and colonic-isolated IBD subtypes CCD and UC (Fig. 2G, Supplementary Figure S3E). *F. prausnitzii* is highly sensitive to bile acids and its absence has previously been used to distinguish ICD [30, 31]. In contrast to *Blautia*, we observed *F. prausnitzii* abundance was decreased in ICD while displaying the same negative correlation with tri- and tetra-hydroxylated bile acids (*p* < 0.05, mean correlation coefficient = – 0.51). This suggests the underlying rationale for the correlation is possibly due to bile sensitivity, not utilization; however, both hypotheses remain to be confirmed (Fig. 3H).

Lastly, we used selected results increased in CCD or ICD to test their performance as potential biomarkers. Nine features that represented the biological classes differentiating ICD and CCD were chosen and their ability to predict these classes (Supplementary Figure S3F). Individually, the sensitivity and specificity of these features varied substantially, ranging from ROC-generated AUCs of 0.63 to 0.84, with proteins having greater accuracy than microbial or metabolic features. When these features were combined in a model, leave-one-out cross validation revealed an 84% accuracy. Test set prediction revealed a slightly greater accuracy (90%) and an AUC of 0.94 (Supplementary Figure S3G). However, unlike our prior models (Fig. 1F), these results are biased by ‘information leakage’ and are thus their performance in a naïve cohort remains to be observed.

Together, multiple lines of evidence suggest ICD’s altered microbial community composition may be largely influenced by increased bile acid levels, with the loss of beneficial microbes such as *F. prausnitzii*, resulting in increased levels of *Gammaproteobacteria* and *Blautia* sp.

### Determination of location-specific disease severity correlates

**Fig. 4:**
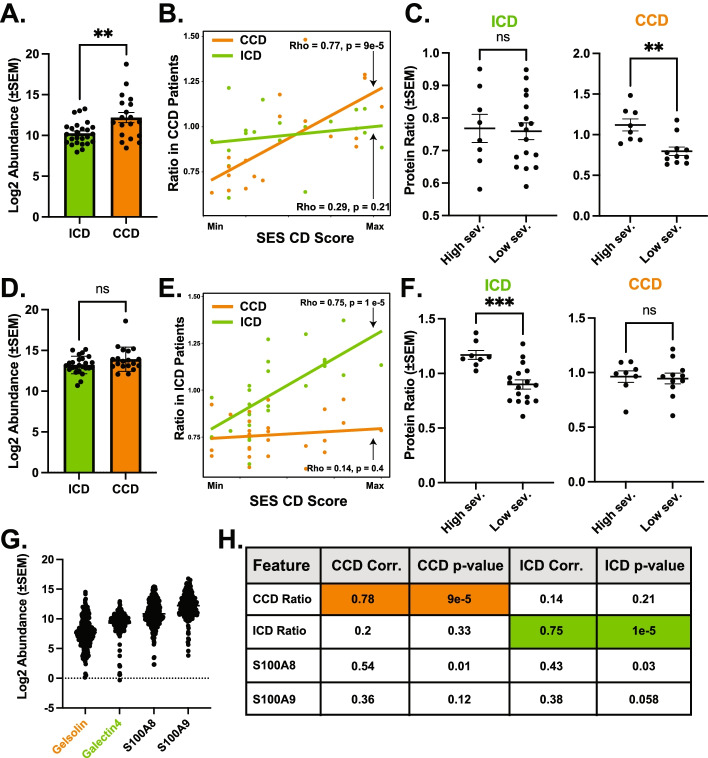
CD location-specific protein pairs outperform calprotectin for inferring severity. **A** Log2-transformed Gelsolin protein abundance comparison between ICD (*n* = 25) and CCD (*n* = 19) abundance. ** denotes *p* < 0.01, Welch’s *t* test. **B** Scatterplot comparing CCD ratio (Gelsolin/Clostridia) pair value and SES-CD disease severity for ICD (ρ = 0.29, *p* = 0.21) and CCD (ρ = 0.77, *p* = 9e^−5^). Ratio input was log_2_-scaled prior to ratio. Correlation values were obtained using Spearman’s correlation. SES-CD scores (*X*-axis) are scaled for visualization purposes. **C** Binned comparisons of CCD ratio pair abundance in ICD and CCD patients binned by “low” and “High” severity (median SES-CD split). ICD high sev. *n* = 8 and low sev. *n* = 17, CCD severity groups high patient *n* = 8, low patient *n* = 10. Statistical comparison performed using Welch’s *t* test, **p = 0.0033. **D** Log2-transformed Galectin-4 protein abundance comparison between ICD and CCD abundance. “NS” = non-significant difference (*P* > 0.05, Welch’s *t* test). **E** Scatterplot comparing ICD ratio pair value and SES-CD disease severity for ICD (ρ = 0.75, *p* = 1e^−5)^ and CCD (ρ = 0.14, *p* = 0.21). Ratio input was log_2_-scaled prior to ratio. Correlation values were obtained using Spearman’s correlation. SES-CD scores (*X*-axis) are scaled for visualization purposes. **F** Binned comparisons of Galectin-4 abundance in ICD and CCD “low” and “high” severity median split groups. ICD severity groups split between high *n* = 8, low *n* = 17 and CCD severity groups split between high = 8, low = 10. Statistical comparison performed using Welch’s *t* test, ***p = 0.0002. **G** Log2-transformed feature abundance for all features used in ratios along with both Calprotectin subunits (S100A8 and 9). All samples (182) used to illustrate broad pattern of expression in comparison to calprotectin expression. **H** Table of statistics comparing the performance of location-specific ratios and calprotectin subunits. All statistics derived using Spearman correlational analysis

Our results confirm ileal and colonic CD subtypes exhibit unique molecular fingerprints, reflecting markedly different pathologies. For instance, neutrophil proteins were less associated with severity in ICD. In line with this, prior work has observed ICD patients’ level of neutrophil-generated fecal Calprotectin, a commonly used inflammatory biomarker, is significantly less useful as a diagnostic marker in ICD compared to CCD [32, 33]. This suggests location-specific biomarkers would facilitate more specific, sensitive, and non-invasive disease severity monitoring. To identify potential location-specific severity biomarkers, we identified proteins most correlated with disease severity in isolated CD subtypes. Furthermore, we reasoned since we selected proteins due to their presence in all samples (including controls), the resulting correlations would likely be reflective of their performance in a larger cohort. We identified Gelsolin, an actin-binding protein, as both highly correlated with CCD disease severity (SES-CD, Spearman ρ = 0.77, *p* = 1e^−4^) and not ICD severity (Spearman ρ = 0.2, *p* = 0.33). Protein abundance of Gelsolin was significantly increased in colonic-isolated IBD subtypes compared to ICD and healthy controls (Fig. 4A, Supplementary Figure S4A). To further enhance the performance of this correlation, we combined Gelsolin and a protein of unknown function from the species *Clostridiales* bacterium (strain VE202-26) that negatively correlated with severity in CCD to generate a ‘CCD severity ratio’ pair (Fig. 4B). Using this ratio resulted in similar correlation scores but nearly 100-fold greater significance and increased disease location specificity (Spearman ρ = 0.78, *p* = 9e^−5^, ICD Spearman ρ = 0.14, *p* = 0.48). Binning patients by the median severity score into two groups (mild vs. severe), ratios of our protein pair maintained the ability to discriminate severity in CCD but not ICD (Fig. 4C). Using the same logic, we generated an ‘ICD severity ratio’ comprised of a protein of unknown function a *Bacteroides* sp. (numerator) and Galectin-4 (denominator). Comparing relative abundance of Galectin-4 in ICD and CCD revealed no significant difference between each group, suggesting its abundance alone did not discriminate between the two conditions (Fig. 4D). Using this ratio resulted in a similarly strong and selective disease score correlation (ICD Spearman ρ = 0.75, *p* = 1e^−5^; CCD Spearman ρ = 0.29, *p* = 0.21) (Fig. 4E). Similar to previous results, binning ICD patients into mild and severe subsets revealed the ICD ratio discriminated between severity in ICD patients but not CCD (Fig. 4F).

We next compared the proposed biomarker ratios’ performance to that of Calprotectin, a canonical severity biomarker commonly referenced in IBD literature. Uncorrected mass spectrometry intensity revealed Calprotectin subunits were on average 5–10× more abundant than either of the proposed host biomarkers, but all were present in our ‘core’ proteome present in all samples (Fig. 4G). Both calprotectin subunits (S100A8-9) were strongly correlated with each other (Spearman ρ = 0.957), confirming the integrity of the measurements. Calprotectin subunit 1 (S100A8) abundance was correlated with both ICD and CCD disease severity, however both significance and magnitude of correlation were appreciably less than the candidate pairs, while subunit 2 (S100A9) did not significantly correlate with either CD subtype (Fig. 4H). We attempted to confirm these results in prior studies but either the proteomes lacked the depth necessary to identify these proteins in untargeted mass spectrometry runs (in uploaded results), or the necessary metadata was not available. Despite this lack of external confirmation, these data suggest the simple protein ratio pairs may serve as highly specific location-based CD severity biomarkers.

## Discussion

Research in past decades has begun to elucidate how genetic mutations, sex, environments, and microbial composition affect CD phenotypes and treatment outcomes. While disease location is a major aspect of CD, it has largely been ignored by -omic studies. As a result, factors influencing location profiles, and thus the underlying pathology, are far less contextually understood. Here, we leveraged an expansive multi-omic IBD cross-sectional study allowing for highly granular analyses relative to smaller studies that lose statistical power comparing IBD subtypes that can easily connect features from disparate technologies. While the feature-sets we leveraged in our targeted analyses of ICD and CCD all displayed reasonable discriminatory power, SNP panels did not. This may suggest that as global profiling tool they provide little utility, or that the study was not large enough for effects to be adequately revealed. It may also mean that their utility lies in the effects individual SNPs impart on host responses and microbial compositions, both questions that deserve additional in-depth analyses that are beyond the targeted scope of our results.

We revealed the molecular uniqueness of ICD and CCD, an important consideration as clinical treatment moves towards the goal of precision medicine [1]. Our results suggest that despite UC and CCD differing in clinical and histological presentation, neutrophil involvement consistently differentiated colonic-isolated subtypes compared to ICD, even when controlling for disease severity. This comports with prior findings in biopsies observing increased expression of host genes associated with neutrophil recruitment in CCD biopsies but not ICD [12]. Deoxyribonuclease-sensitive perinuclear anti-neutrophil cytoplasmic antibodies, a common marker for neutrophil activity, were also more likely to be present in CCD subjects [34]. Neutrophil-secreted products were also more correlated with severity in CCD subjects compared to ICD counterparts, echoing and expanding previous results [32]. Lastly, prior proteomics profiling of CCD and ICD biopsy differences also concluded that CCD biopsies also harbored greater abundances of neutrophil-related proteins [35]. Importantly, this controls for the extent of tissue assayed, which is a significant limitation of stool-based assays, as they can collected proteins from all along the intestinal tract, possibly skewing measurements. Regardless, a more direct and controlled comparison of differential neutrophil activity in CCD and ICD is needed to confirm that this is not solely the result of extended tissue involvement and is reflective of differential pathology. We noted increased colonic neutrophil-related proteins were paired with the increased presence of amino acids and dipeptides, potentially the result of increased proteolytic activity in the colon. Prior research has established abnormal proteolytic activity as a hallmark of IBD [36]. Our lab, and others, identified *B. vulgatus* protease levels were strongly associated with UC inflammation severity [15, 37]. While the host- and microbe-driven proteolytic phenotype has yet to be confirmed in CCD using orthogonal assays, we found *B. vulgatus* to be associated with increased CD severity solely in CCD, and further correlated with proteolytic products such as dipeptides. However, it must be noted that neutrophils and other immune cells also secrete many potent proteases associated with increased disease burden. Therefore, the degree to which hosts and microbes contribute to pathologic proteolytic environments in colonic diseases remains to be determined by future studies. These future studies should also consider combining untargeted multi-omic studies with more targeted assays such as cytokine profiling to more fully characterize immune networks involved. This is critical, as cytokines, due to their low abundance, are virtually never identified in stool without the aid of enrichment techniques.

With regards to ICD, our analyses identified several key taxa with noted bile acid associations, suggesting the altered bile acid levels found in ICD patients may ultimately shape their microbial communities and influence downstream pathology, possibly due to the loss of beneficial byproducts such as short-chain fatty acids. In line with this, the loss of *F. prausnitzii* in ICD patients is a well-known phenomenon and has been characterized as part of the “F-E” index (*F. prausnitzii* + *E. coli*) used to discriminate between IBD subtypes [38]. Here, we expand the list of microbes discriminating ICD and CCD with results suggesting *Blautia* sp. (often *B. obeum*)*,* and *Lachnospiraceae* in general, are more abundant in ICD. Prior results found *Blautia* decreased broadly in CD; however, to our knowledge, we report this loss is potentially isolated to CCD [39]. *Blautia’s* noted ability to process primary bile acids further cements its role in the bile-driven ICD ecosystem. Future studies should consider targeted studies of bile acid sensitive- and resistant-species and the effect various types of common bile acids have on their abundance and functional profiles. Considering results distinguishing both ICD and CCD, it is possible that a major driver in gut-microbe composition may be their greater sensitivity to host immune products or the antimicrobial effects of bile acids.

Given the clear profile differences between isolated ileal and colonic CD, we leveraged our feature set to create novel, location-specific disease biomarker pairs. In our cohort, the selected markers more accurately predicted disease severity than either Calprotectin subunit. We further confirmed the proteins used were readily detectable in every stool sample collected (including healthy), albeit at lower levels than those of Calprotectin subunits. Despite this, the use of highly sensitive detection techniques such as ELISA would likely overcome this deficit given their ability to detect extremely low-abundant biomarkers such as cytokines (virtually never identified in untargeted shotgun proteomics feature sets). If confirmed in a new cohort and developed into a point-of-care assay, these biomarker pairs (even in singular non-pair, in the case of host proteins, for the purposes of greater consistency in a wide population) could help monitor disease without the use of colonoscopies.

Despite these promising findings, limitations on their applicability remain. While our cohort was large compared to prior studies, moving toward reliable non-endoscopic disease diagnostics via machine learning will require thousands of samples selected meticulously to answer a specific question. Unfortunately, this requirement is not often feasible in clinical settings where most patients vary in many dimensions. This underscores the need for a stool-omics field-wide effort to establish standardized processing and collection protocols, allowing for the integration of multiple smaller cohorts. An additional limitation of this study was missing feature annotations, a common issue in -omics studies. Indeed, despite the use of custom metagenomic-generated proteome databases, and machine-learning-based molecular networking for metabolite identification, many features were of little value due missing or unhelpful (e.g., “hypothetical protein” or “unknown metabolite”) annotations, hindering our ability to fully characterize location profiles. Thus, future cohort profiles would undoubtedly benefit from a more robust annotation pipeline including targeted strain-level metagenomic searches for microbes, and spike-in panels for important metabolite families (e.g., bile acids) that emerge after initial analyses. While this would necessitate additional runs, the information gleaned would be much more impactful. Lastly, due to stool’s intestinal transit, it is difficult to identify the origin of some proteins and microbes. For instance, the presence of increased myosin-related proteins in ICD patients seems useful as a general biomarker but does not provide evidence for its source along the intestinal tract. Moreover, stool is somewhat biased towards resembling colonic microbial communities [40]. Conversely, many proteins have noted sources, such as digestive enzymes and neutrophil related proteins, suggesting stool has a mixed capacity for localization, which could be addressed in more targeted studies. Despite these challenges, stool-based -omics studies line up well with more spatially targeted studies, and lead to real mechanistic insights [15, 35].

In summary, by leveraging a large IBD cohort combined with extensive multi-omic profiling, our analyses present both novel and confirmatory insights on IBD subtypes and further establish the utility of multi-omic strategies for identifying biomarkers. The extensive profiles allowed for highly granular subgroup analyses, providing strong evidence for the decoupling of ICD and CCD despite some commonalities. Given the evidence that ICD and CCD host and microbial profiles differ in a biologically consequential manner, the next steps must consist of greater understanding of mechanistic underpinnings. Achieving this goal will bring us one step closer to potential therapeutic paradigm shifts in the field of CD treatment.

## Conclusions

Despite both being categorized as CD, our analyses highlight the utility of stool-based multi-omics for the elucidation of disparate mechanisms influencing ICD and CCD pathology. CCD’s profile highlights the integral role host inflammation and proteolytic activity play in disease severity, while ICD is characterized by changes in the abundance of bile acid-resistant and sensitive microbial constituents. These findings drive the identification of proteins that may be useful for the non-invasive monitoring of disease severity.

## Methods

### Patient demographics and disease severity scoring

Patient demographics (age, gender, ethnicity), disease history (prior surgeries, complications, Montreal sub-classifications), current and prior therapies (corticosteroids, immunomodulators, biologics), patient-reported disease activity (partial Mayo and CDAI), and endoscopic and histologic disease activity (SES-CD, UCEIS, Mayo) were recorded. Endoscopic scoring was done by a physician blinded to any information pertaining to study. Paired stool samples and endoscopic assessments were done within 24 h. Further details regarding clinical metrics and endoscopic and histologic activity scoring are discussed further in Dulai et al. [41].

### Isolation of stool proteins and peptides

Peptides from stool samples were isolated as described by Mills et al. Briefly, 0.5 g of stool was suspended in TBS. Particulate was removed using steriflip (Milipore) filters. Cells were suspended in a 4-mL mixture of lysis buffer and 4M urea and lysed via probe sonication. Proteins were reduced and alkylated, then precipitated using chloroform and methanol. Peptides were generated by digesting overnight with LysC (Wako), and a 6-h trypsin digestion at 37 °C. Peptides were desalted using C18 Sep-Paks (Waters). Tandem Mass Tag (TMT, Thermo Fisher Scientific) 10 plex kits were used with a dedicated channel used containing a study-wide representative peptide mixture used for normalization between runs.

### Metaproteomic data collection and processing

Data collection and processing was performed as described by Mills et al. [42] In brief, combined peptides from each TMT experiment underwent offline basic pH reverse-phase liquid chromatography (LC) using C18 columns on an Ultimate 3000 HPLC (Thermo Scientific), separating each experiment into 24 fractions. Twelve fractions underwent LC-MS2/MS3 analysis on an Orbitrap Fusion mass spectrometer (Thermo Fisher Scientific) utilizing in-line fractionation for 60 min on an Easy-nLC 1000 (Thermo Fisher Scientific).

Data was processed using Proteome Discoverer 2.1 (Thermo Fisher Scientific), with MS2 spectra searched against a custom in-house database of microbial proteins identified from the metagenomic sequencing analysis, and the Human proteome (uniport.org, accessed 5/11/2017). Search parameters were set as previously described, and data was quality controlled at a 1% false discovery rate for both peptide and protein identifications.

### SNP feature generation

Genomic DNA was extracted from patient blood using DNeasy DNA extraction columns (Qiagen). Purified DNA was then quantified using a Qubit 4 fluorometer (Thermo Fisher Scientific) and aliquoted to 30 μg/μL. Samples were then processed further and amplified and genotyped using Illumina Infinium Global Diversity Array (Illumina, Inc.). Data acquired was then processed using Illumina Genom Studie v2. Prior to output, we applied the following settings: Call freq. ≤ 0.99, Cluster separation < 0.45, AA R mean, < 0.4, AB R mean < 0.4, BB R mean < 0.4, 10% GC score ≤ 0.3, heat excess > 0.2, AB freq. ≥ 0.4, AB t. mean < 0.2 or > 0.8. Statistics on the resulting data were done using the R package snpReady, and alleles were encoded numerically as follows: AA = 0, Aa = 1, and aa = 2, following snpReady package instructions. These features were further plotted as a PCA using FactoExtra R package.

### Generation of sequencing data sets

Shotgun sequencing data (EBI Project Identifier PRJEB42155) was mapped to the web of life microbial genome database using Centrifuge 1.0.3 with default parameter settings [43]. Reads were summarized per reference genome per sample. Genomes mapped by less than 0.01% reads per sample were dropped. 16S sequencing data (EBI Project Identifier PRJEB42155) was split demultiplexed, trimmed to 150 bp and assigned to amplicon sequencing variants via deblur using [44].

### Generation of metabolomic feature set

Samples and data was processed as previously described [15]. In brief, stool samples were weighed, and metabolites were extracted at a 1:5 ratio of wet weight from fecal material to 70% methanol infused with 5 μM internal standard sulfamethoxine, vortex, and left to extract overnight at 4°C. Supernatant extraction was then centrifuged to remove particulate and placed in a 96-well plate and diluted 1:4 with methanol. Shotgun LC-MS/MS was performed on a Bruker Maxis qTOF mass spectrometer (Bruker, Billerica, MA USA) with in-line HPLC fractionation using a ThermoScientific UltraMate 3000 Dionex UPLC (Fischer Scientific, Waltham, MA USA) equipped with a Kinetex C18 column flowing at 0.5 mL/min. Mobile phase gradient was run for 850 s. starting from 98:2 water:acetonitrile to 2:98 water:acetonitrile. MS was run in positive mode (m/z 50-2000) using a data dependent selection of the top ten most intense ions per MS1 scan chosen for MS2 level analysis. Lock mass internal calibration used a wick saturated with hexakis (1H,1H,3H-tetrafluoropropoxy) phosphazene ions (Synquest Laboratories, m/z 922.0098) located within the source.

Individual MS runs were aligned using mzMine software (parameters described previously) [45]. Area under the MS1 peak was utilized for feature abundance values, and two strategies were used for feature identification based on MS2 fragmentation patterns. One method utilized feature-based molecular networking [46], the other method utilized the Qemistree workflow [47]. Both methods were performed utilizing the online metabolomics database and processing servers provided by GNPS [17]. Based on prior literature, quantification values for each sample were subsequently normalized using rarefaction based on minimum intensity of 1e8 [48]. This data was further scaled for integration into the multi-omic feature set.

### Machine learning pipeline

Each dataset was first split 70/30 into two sets (training and testing, respectively), balancing SES-CD scores, location, diseases, and UCEIS scores. Using the training set, we performed feature selection using the R package EFS (v1.0.3). The selected features were then used to subset each full omics set. Using these subsets, each algorithm was trained using the Randomized Search CV package from SciKit-Learn (v0.24.0) with 5-fold cross-validation and targeting the setting ‘balanced accuracy’ to account for imbalanced data sets. We then used the testing set and recorded the accuracy, precision, and recall of the highest scoring algorithm amongst the five used.

Initially 10 different algorithms were tested for the general predictive performance on ICD vs. CCD and CCD vs. UC and CD vs UC: (1*)RandomForest, (2*)ExtraTrees, (3*)Decision Trees, (4*)SVC, (5)MLPC, (6*)Voting Classifier, (7)Naïve Bayes, (8)K-nearest Neighbors, (9)Logistic Regression, (10)Adaboost. *Denotes a classifier that was chosen for all subsequent comparisons. Five were found to produce inconsistent results and were not used further.

To account for algorithmic biases, we performed permutation of feature importance (SciKit-Learn v0.24.0) on the test feature set, which recorded the loss in classification performance after each permutation (*n* = 100).

### Statistical analyses

Where applicable, multivariate statistics were computed using the R statistical program and matrixTests package (v0.1.9), with multiple hypothesis testing added using “false discovery rate” (also known as Benjamini-Hochberg correction) using Hmisc (p.adjust) R statistical package and Qiime2 in the case of groupwise statistics [49]. Univariate statistics were computed using Prism (v9.0.0). Specific statistical tests undertaken are reported in text section or associated figure legend. Songbird analysis was done using the Qiime2 version of software.

## Supplementary Information

**Additional file 1: Supplementary Figure S1.** IBD200 Cohort supplementary metrics A) Severity breakdown of CD and UC cohorts. SES-CD and UCEIS (UC severity scale) used for scoring breakdown. B) Proteomic feature set comparison to previously reported values in literature. Every effort was taken to include reported values from IBD stool metaproteomic studies from the past five years, however this list may not be exhaustive. PubMed search terms used “Inflammatory bowel disease meta/proteome”, “Ulcerative colitis meta/proteome”, “Crohn’s disease meta/proteome”. C) SNP panel generated statistics on 126 IBD patients in cohort. Left panels are histograms of Nei’s genetic diversity and minor allele frequency. Right panel is PCA graph colored by disease location (only ICD n = 27, and CCD n = 22, subtype used in graph generation). Colored circles indicate confidence interval (0.95). D) Comparison of discriminatory power of each feature set, as calculated by Log2(Pseudo-F/P-value). Both input values determined by PERMANOVA beta-diversity comparisons (see Supplementary Table 1 for extended results). E) PCoA biplot of multi-omic feature set. Top 10 features are plotted in biplot, colored by active IBD location subgroup, please refer to Supplementary Table 3 for details.

**Additional file 2: Supplementary Figure S2.** Features distinguishing CCD from ICD A) Enrichr plot generated from severity-controlled comparison of CCD to ICD (q-value = 8e-11). Of note, given the greater number of segments that can be scored for severity in CCD, this comparison is for presentation purposes only and does not truly represent a fair comparison to ICD (e.g. ICD patients could have more severe inflammation and still not score as high as CCD with less inflammation). B) Comparison of neutrophil-SES-CD score Spearman correlations in two single segment CD subtypes (ICD and rectal-only inflammation). Only significantly correlated proteins (p < 0.05, ICD = 17 proteins, Rectal = 14 proteins) were used in comparison. **** = p < 0.0001, Welch’s t-test. C) Comparison of neutrophil-SES-CD score Spearman correlations in two single segment CD subtypes (ICD and rectal-only inflammation) further controlled for SES-CD score (ICD mean = 6, CCD = 6.5). Top 10 most highly correlated proteins used. * = p < 0.05, Welch’s t-test. D) String-DB-generated network of combined inflammation-related cytokines and other proteins, filtered for high-confidence interactions (interaction score ≥ 0.7, see Supplementary Table 4) and proteins significantly increased in CCD compared to ICD. Grey dots represent imputed features, orange is features increased in CCD. E) Heatmap of mean dipeptide abundance per CD subtype. F) Plot of Log_2_ ratios of *B. vulgatus* in CD subtypes. No significance noted (ANOVA adjusted p > 0.05). ICD n = 25, CCD n = 17, ICCD n = 8. G) Plot of ICD and CCD Spearman correlation values generated by comparing *B. vulgatus* proteases (metaproteome features, log2-transformed) and SES-CD score. Welch’s t-test, *p* = 0.06.

**Additional file 3: Supplementary Figure S3.** Information supporting the distinction between ICD and CCD A) Comparison of mean abundance of Myosin-isoform proteins between ICD patients with either penetrating or structuring wounds (n = 10) to those with no wounds present (but still active disease, n = 9). * p < 0.05, Welch’s t-test. B) String-DB-generated network of combined inflammation-related cytokines and other proteins, filtered for high-confidence interactions (interaction score ≥ 0.7, see Supplementary Table 4) and proteins significantly increased in ICD compared to CCD. Grey dots represent imputed features, green is features increased in ICD. C). Expanded heatmap of features from the genus *Blautia* (x-axis) split by CD subtype (right) and further color coded by disease severity. See section 1 results for disease scoring split. Feature abundance scaled by column. D) Spearman correlation measurements comparing different bile acid families and SES-CD scores. Each dot represents mean correlation of a bile acid (or subtype) and metagenomic *Blautia sp*. features. E) Abundance of *F. prausnitzii* features from both metaproteome (left and middle) and ASV (right) feature sets in CD subtypes. * p < 0.05, *** p < 0.001, Welch’s t-test. F) ROC curve of individual features differentiating ICD and CCD. Features were chosen due to their statistical significance and representation of classes explored in the results. G) ROC curve generated by a model trained (ExtraTrees classifier) on all features in Supplementary Fig. 3F, and tested using a 70/30 split, using Leave-one-out cross validation.

**Additional file 4: Supplementary Figure S4.** Information supporting the distinction between ICD and CCD A) Log2 scaled abundance of Gelsolin among various categories in study. Multiple comparison statistics performed using Brown-Forsythe and Welch ANOVA, * = p < 0.05, ** = p < 0.01, *** = p < 0.0005.

**Additional file 5.**

**Additional file 6.**

**Additional file 7.**

**Additional file 8.**

**Additional file 9.**

**Additional file 10.**

## Data Availability

R and Jupyter notebook scripts are freely available at https://www.github.com/c6gonzalez/IBD200/ and a copy has been attached as supplementary information for review purposes. Raw metabolomics data is available at https://massive.ucsd.edu under study ID MSV000084908. Metaproteome specific search considerations were undertaken as previously described [42]. Raw data as well as processed data tables are available online at https://massive.ucsd.edu under study identifier MSV000086509. Shotgun metagenomic and 16S rRNA feature sets are deposited in QIITA (Study ID 11549, https://qiita.ucsd.edu/).

## Abbreviations

IBD: Inflammatory bowel disease
CD: Crohn’s disease
UC: Ulcerative colitis
ICD: Ileal Crohn’s disease
CCD: Colonic Crohn’s disease
ICCD: Ileocolonic Crohn’s Disease
SES-CD: Simple endoscopic score-Crohn’s disease
UCEIS: Ulcerative colitis index of severity
CDAI: Crohn’s disease activity index
PCoA: Principle coordinate analysis
ANXA3: Annexin A3
MYH2: Myosin-2
ROC: Receiver operator curves
PR: Precision recall curves
MTG: Metagenome
MTB: Metabolome
MTP: Metaproteome
16S: 16S rRNA ASVs
OGU: Operational genomic unit
ASVs: 16S Amplicon sequence variants
TMT: Tandem mass tags
